# Invadopodia are chemosensing protrusions that guide cancer cell extravasation to promote brain tropism in metastasis

**DOI:** 10.1038/s41388-018-0667-4

**Published:** 2019-01-16

**Authors:** Karla C. Williams, Mario A. Cepeda, Sumreen Javed, Karlee Searle, Katie M. Parkins, Ashley V. Makela, Amanda M. Hamilton, Sepideh Soukhtehzari, Yohan Kim, Alan B. Tuck, John A. Ronald, Paula J. Foster, Ann F. Chambers, Hon S. Leong

**Affiliations:** 1 Department of Surgery, Division of Urology, Schulich School of Medicine and Dentistry, Western University, London, ON Canada; 2 Translational Prostate Cancer Research Laboratory, Lawson Health Research Institute, London, ON Canada; 3 Translational Breast Cancer Research Unit, Lawson Health Research Institute, London, ON Canada; 4 Faculty of Pharmaceutical Sciences, University of British Columbia, Vancouver, BC Canada; 5 Department of Urology, Mayo Clinic, Rochester, MN USA; 6 Department of Medical Biophysics, Schulich School of Medicine and Dentistry, Western University, London, ON Canada; 7 Robarts Research Institute, Western University, London, ON Canada; 8 Departments of Oncology, and Pathology and Laboratory Medicine, Schulich School of Medicine and Dentistry, Western University, London, ON Canada

**Keywords:** Metastasis, Cytoskeleton

## Abstract

Invadopodia are cell protrusions that mediate cancer cell extravasation but the microenvironmental cues and signaling factors that induce invadopodia formation during extravasation remain unclear. Using intravital imaging and loss of function experiments, we determined invadopodia contain receptors involved in chemotaxis, namely GABA receptor and EGFR. These chemotaxis capabilities are mediated in part by PAK1 which controls invadopodia responsiveness to ligands such as GABA and EGF via assembly, stability, and turnover of invadopodia in vivo. PAK1 knockdown rendered cells unresponsive to chemotactic stimuli present in the stroma, resulting in dramatically lower rates of cancer cell extravasation and metastatic colony formation compared to stimulated cancer cells. In an experimental mouse model of brain metastasis, inhibition of PAK1 significantly reduced overall tumor burden and reduced the average size of brain metastases. In summary, invadopodia contain chemotaxis receptors that can respond to microenvironmental cues to guide cancer cell extravasation, and when PAK1 is depleted, brain tropism of metastatic breast cancer cells is significantly reduced, blocking secondary colony growth at sites otherwise permissive for metastatic outgrowth.

## Introduction

Metastasis is a complex and multistep process in which tumor cells released by a primary tumor go on to form secondary colonies at distant sites via the hematogenous and lymphatic circulatory system. This process involves tumor cell migration away from the primary site, then entry into (intravasation) and out of (extravasation) the bloodstream, followed by subsequent survival and growth at a secondary site. Despite being an inefficient process, metastasis will eventually lead to colonization of distant vital organs, leading to cancer patient mortality [1, 2]. The acquisition of an invasive phenotype is critical for successful cancer cell dissemination and is mediated in part by invadopodia at key steps of metastasis such as extravasation [3]. Invadopodia have been heavily studied in vitro and in vivo, providing crucial insights into the therapeutic potential of targeting these cancer-specific protrusions to prevent metastasis [4–7].

Invadopodia are dynamic actin-rich protrusive structures capable of degrading the extracellular matrix [8]. Their formation has been demonstrated to occur in response to growth factor stimulation [9] and extracellular matrix-mediated integrin signaling [10]. Signaling by these growth factors and integrins stimulates localized F-actin nucleation which is modulated by actin regulatory proteins such as N-WASP, cortactin, and ARP2/3 which then assist in invadopodia formation [11, 12]. Other key regulatory proteins such as TKS5 play a role in invadopodia maturation and degradation of the extracellular matrix [13–15]. Protein trafficking to the membrane is also important in the formation and maturation of invadopodia because of the delivery of proteins such as integrins and MT1-MMP [16–19]. These events result in the protrusion and elongation of invadopodium into the extracellular space, followed by a turnover event where the plasma membrane is uncoupled from actin.

Formation of invadopodia is well characterized, but less is known about the molecular cues that prompt invadopodia assembly and disassembly. Currently, it is understood that paxillin phosphorylation in invadopodia/podosomes can regulate disassembly through ERK and calpain activation [20, 21], which can be regulated by the small GTPases RhoG [22]. Turnover of invadopodia is also known to involve Rac1 and the downstream effector p21-activated kinase 1 (PAK1), which phosphorylates cortactin on Ser113, causing its release from F-actin [23]. p27^Kip1^ regulates cortactin binding and activation by PAK1 regulating invadopodia stability and turnover [24]. These studies have enhanced our understanding of disassembly in vitro, however, when and why invadopodia disassembly occurs has yet to be elucidated.

This study evaluated the in vivo role of PAK1 in invadopodia function and turnover, and identified additional “outside-inside” signaling pathways regulating disassembly. We have examined PAK1 regulation of cofilin and myosin light chain (MLC) phosphorylation at invadopodia, as well as the in vivo effects of blocking invadopodia dissolution through PAK1 knockdown. We found that invadopodia disassembly in response to chemotactic factors, as mediated by PAK1, is important for directing cancer cell extravasation in microenvironments and is a worthy anti-metastasis target.

## Results

### PAK1 regulates invadopodia disassembly in invasive cancer cells

**Fig. 1:**
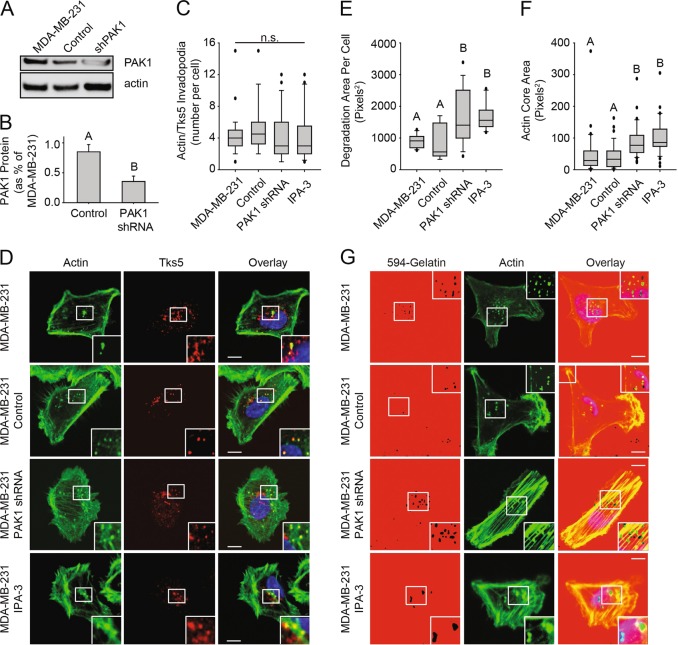
PAK1 regulates actin core dynamics and invadopodia disassembly. MDA-MB-231 cells were either untreated, or lentiviral infected with control or PAK1 shRNA to generate stable knockdowns, or treated with 1 µM IPA-3. **a** Representative Western blot of PAK1 levels in MDA-MB-231, control and PAK1 shRNA cells. **b** Quantification of Western blot experiments in. Means ± S.E. (error bars) from three independent experiments are shown. Student’s *t*-test was used to determine significant differences. Different letters denote a value significantly different from MDA-MB-231 cells (*p* < 0.05). **c**, **d** Cells were plated on gelatin-coated coverslips for 3 h, fixed, permeabilized, and stained using anti-TKS5 antibody, followed by Alexa594-conjugated secondary antibody and Alexa488-phalloidin to stain F-actin. **d** Single confocal slices of the ventral surface of cells are shown. Areas of colocalization are seen in the overlay as yellow. **c** Quantification of TKS5 and F-actin-containing punctae were counted using confocal microscope. Means ± SEM from 3 independent experiments in which 20–30 cells per sample were assessed are shown. A two way ANOVA comparing all four groups showed no significance (n.s.). **e**–**g** Cells were plated on Alexa594-labeled gelatin for 5 h. GM6001 was added for 3 h, washed out and cells were fixed 2 h later. **e** Invadopodium-based degradation of the 594-gelatin matrix of individual cells was quantified using ImageJ software. Means ± SEM from 3 independent experiments in which 10–20 cells per sample were measured are shown. A two way ANOVA was used to determine significant differences. Different letters denote significant differences (*p* < 0.05). **f** Actin core size at invadopodium-based degradation sites of individual cells was quantified using ImageJ software. **g** Confocal microscopy images at the ventral cell surface showing spots of invadopodium degradation (black holes) with overlaying actin cores (green). Means ± SEM from 3 independent experiments in which 10–20 cells per sample were measured are shown. One-way ANOVA was used to determine significance. Different letters denote significant differences (*p* < 0.05). Scale bar = 10 µm

PAK1 has been shown to play a role in invadopodia disassembly in breast carcinoma cell lines [23] and it has also been suggested to regulate invadopodia formation in melanoma cells [25]. To investigate the role of PAK1 in invadopodia formation and disassembly we generated shRNA knockdowns of PAK1 in the breast cancer cell line MDA-MB-231 (shPAK1). Knockdown of PAK1 was confirmed through Western blotting, revealing a knockdown efficiency of 95 ± 3% (Fig. 1a, b). To investigate if PAK1 regulates invadopodia formation, the number of invadopodia per cell was quantified based on the colocalization of established invadopodia markers TKS5 and actin. No significant differences were found between control and PAK1 knockdown cells (Fig. 1c, d). Impairing PAK1 activity using a selective allosteric PAK1 inhibitor IPA-3 to perturb PAK1 phosphorylation [26] also showed no significant differences in the number of invadopodia per cell (Fig. 1c, d). In addition, we assessed the ability of PAK1 knockdown and IPA-3 treated cells to degrade the extracellular matrix (ECM) and no change was observed in the percentage of cells that formed functional invadopodia (Supplemental Fig. 1). However, PAK1 knockdown cells and IPA-3 treated cells degraded on average 74.7% and 82.1%, respectively, more ECM compared to control cells (*P* = 0.016) (Fig. 1e, g). Since PAK1 activity can regulate cytoskeletal events such as focal adhesion turnover and actin rearrangements [27–29], we hypothesized that the regulation of actin dynamics by PAK1 could also influence invadopodia formation. To assess this, actin core size at sites of invadopodium degradation were quantitated and found to be on average 2.3 and 2.6 fold larger in PAK knockdown and IPA-3 treated cells, respectively, compared to control (*p* < 0.001) (Fig. 1f, g). Since impairing PAK1 did not increase the number of total invadopodia formed, but rather altered the size of these structures, this suggests that PAK1 may regulate the lifetime of invadopodia by influencing actin core dynamics.

To determine if these results were specific to MDA-MB-231 cells, we generated PAK1 knockdowns in the brain-metastatic cell line MDA-MB-231BR and the metastatic HER2 positive breast cancer cell line 21MT-1 (Supplemental Fig. 2A and B). Observations regarding PAK1 knockdown were consistent across all cell lines with a significant increase in invadopodium degradation and actin core size but with no change in the number of invadopodia per cell (Supplemental Fig. 2C, D and E). To determine if PAK1-regulated disassembly is exclusive to breast cancer cells, PAK1 was also knocked down in the renal cell carcinoma cell line 786-0 (Supplemental Fig. 2F), revealing similar results in carcinoma of a different disease site, suggesting that invadopodia regulation by PAK1 is conserved across various cancer cell types (Supplemental Fig. 2G and H). These results corroborate published findings from [23] showing that PAK1 regulates invadopodia lifetime by mediating disassembly.

### PAK1 regulates cofilin and myosin light chain phosphorylation at invadopodia

**Fig. 2:**
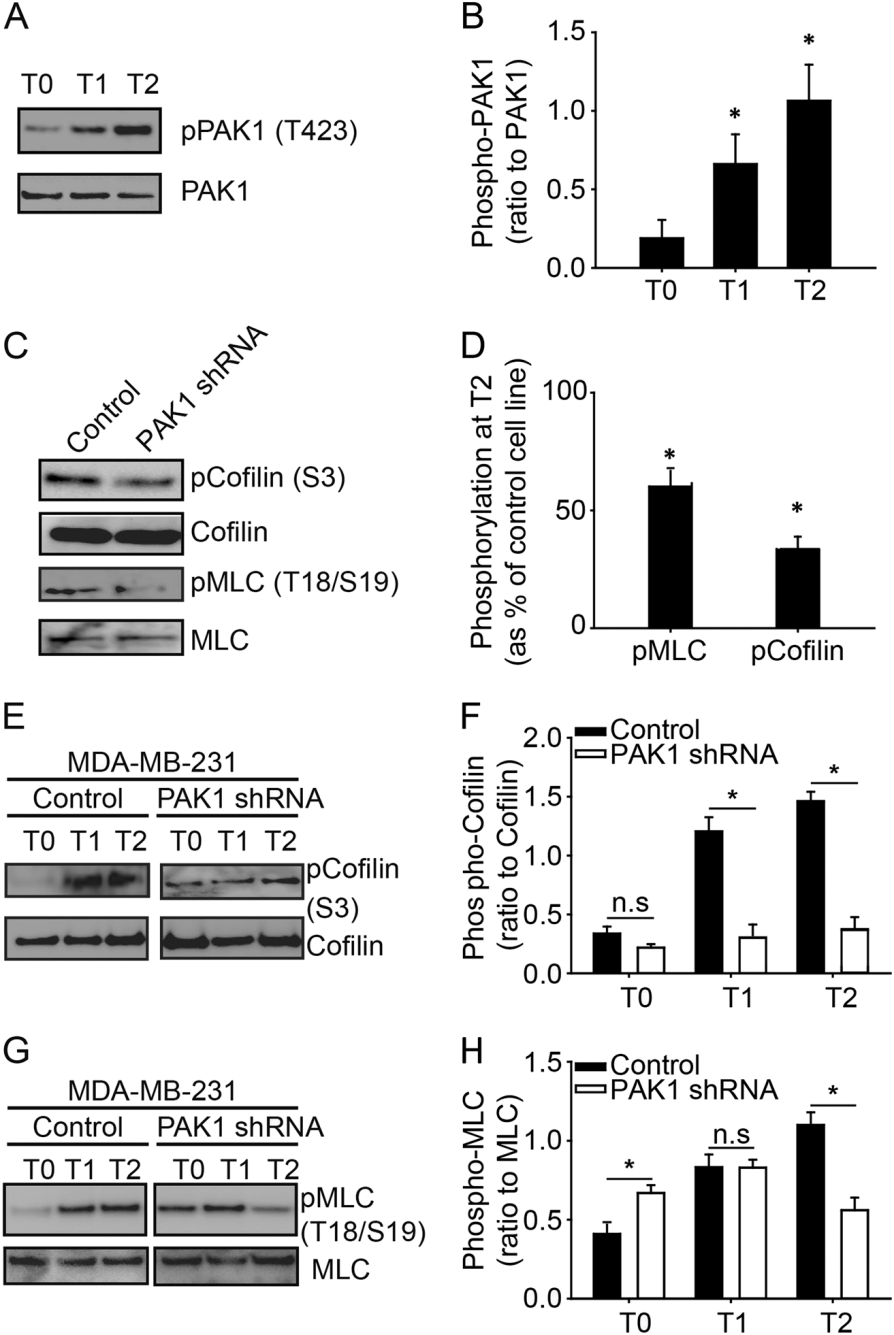
PAK1-mediated phosphorylation of cofilin and myosin light chain during invadopodia formation and disassembly. MDA-MB-231 control and PAK1 shRNA cells were plated on 0.2% gelatin plates with 25 µM GM6001 for 3 h, rinsed, and either lysed (T0), or media was replaced and cells were incubated for 1 h (T1) or 2 h (T2). **a** Representative western blot of PAK1 Thr423 phosphorylation levels. **c** Representative western blot of phosphorylated cofilin Ser3 and myosin light chain Ser18/Thr19 at T2 compared to control. **e**, **g** Representative western blot of phosphorylated cofilin Ser3 and myosin light chain Ser18/Thr19 during invadopodia formation, T0 and T1, and disassembly, T2. **b**, **d**, **f** and **h** Quantification of western blot experiments in (**a**), (**c**), (**e**), (**g**). Means ± S.E. (error bars) from three independent experiments are shown. Student’s *t*-test was used to determine significant differences. Asterisk denotes a value significantly different from control cells (*p* < 0.05)

While PAK1 can regulate invadopodia disassembly, in part through cortactin phosphorylation [23], cytoskeletal events regulated by PAK1 are diverse and we sought to further investigate the role of PAK1 in invadopodia disassembly. We investigated the role of PAK1 in the phosphorylation of an established invadopodium regulator, cofilin, and in the phosphorylation of myosin light chain (MLC). To assess phosphorylation levels of PAK1 and its cytoskeletal substrates during invadopodia formation, an established time course invadopodia formation assay was used [30]. Cells plated on gelatin-coated coverslips were initially synchronized through the use of matrix metalloproteinase inhibitor (GM6001) and then washed out (Time point 0, T0), enabling invadopodia maturation and matrix degradation (Time point 1 h, T1), followed by actin core disassembly (Time point 2 h, T2) [30]. PAK1 activity as determined by phosphorylation on Thr423 was assessed during invadopodia formation, maturation and disassembly, revealing a significant increase at T1 and T2 compared to T0 (Fig. 2a, b) (*P* < 0.05). To determine if PAK1 activity was regulating cofilin and MLC phosphorylation, their phosphorylation levels during invadopodia disassembly (T2) were assessed. Both cofilin and MLC phosphorylation were lower in PAK1 knockdown cells compared to control (Fig. 2c, d). This was further confirmed using the selective PAK1 inhibitor IPA-3 which showed that impairing PAK1 activity significantly reduced both cofilin and MLC phosphorylation levels (Supplemental Fig. 3). Next, we assessed PAK1-mediated phosphorylation of MLC and cofilin during invadopodia formation (T0), maturation (T1) and disassembly (T2). Levels of phosphorylated cofilin were consistently reduced in PAK1 knockdown cells across all time points with a significant reduction found at T1 (*P* = 0.040) and T2 (*P* = 0.022). Phosphorylated MLC was significantly increased by 20% at T0 (*P* = 0.029), with no difference observed at T1, and a significant reduction of > 50% observed at T2 (*P* = 0.011) (Fig. 2e–h). Similar results were found in MDA-MB-231BR and 21MT-1 cells (Supplemental Fig. 4). These results demonstrate an oscillating role of PAK1 in MLC phosphorylation, which is corroborated by previous findings showing that PAK1 activity promotes MLC phosphorylation during directed migration and invasion [29, 30] and others showing that PAK1 activity during cell spreading inhibits MLC phosphorylation via myosin light chain kinase activity [27].

**Fig. 3:**
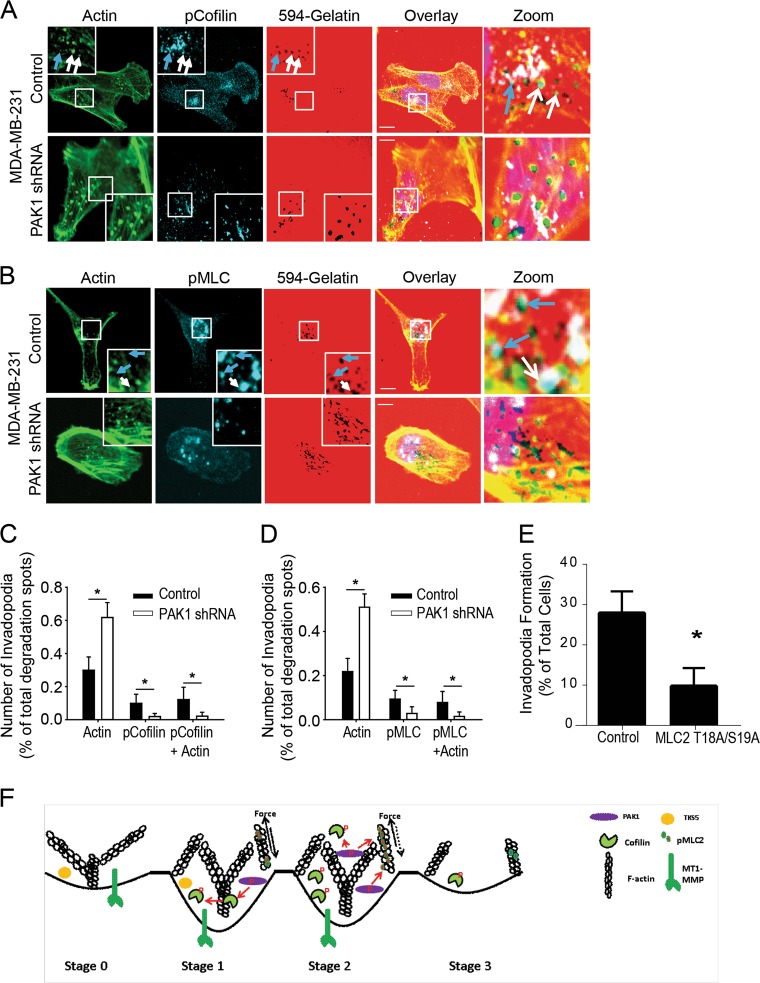
PAK1 regulates myosin light chain and cofilin phosphorylation at invadopodia. MDA-MB-231 control and PAK1 shRNA cells were plated on Alexa594-gelatin coverslips with 25 µM GM6001 for 3 h, rinsed, and either fixed (T0), or media was replaced and cells were incubated for 1 h (T1) or 2 h (T2) fixed, permeabilized, and stained. Cells were stained using anti-pMLC S18/T19 or pCofilin S3 antibody, followed by Alexa647-conjugated secondary antibody and Alexa488-phalloidin to stain F-actin. **a**, **b** Single confocal slices of the ventral surface of cells are shown. Arrows point to areas of colocalization of proteins, with dark spots representing areas of gelatin degradation. pMLC and pCofilin localize at sites of invadopodium degradation with (white arrows) and without F-actin punctae (blue arrows), as seen in overlay. **c**, **d** Quantification of degradation spots with actin cores, or with pMLC ± actin or pCofilin ± actin were counted using confocal microscope. Means ± S.E. (error bars) from three independent experiments are shown in which 10–20 cells per sample were measured are shown. **e** MDA-MB-231 control and MLC2 T18A/S19A cells were plated on Alexa594-gelatin coverslips for 4 h, fixed permeabilized, and stained for F-actin. The percentage of cells forming invadopodia, based on degradation of the fluorescent gelatin, was quantified using confocal microscopy. Means ± S.E. (error bars) from three independent experiments are shown in which cells were quantified as either invadopodia forming or non-forming in 10–20 fields of view. Student’s *t*-test was used to determine significant differences. Asterisk denotes a value significantly different from control cells (*p* < 0.05). Scale bar = 10 µm. **f** Model of PAK1-mediated invadopodia disassembly as regulated through cofilin and MLC activity. At Stage 0 invadopodia have formed but are unable to degrade the matrix due to GM6001 treatment. Upon GM6001 removal, invadopodia mature as they degrade the matrix and extend into the substratum (Stage 1) followed by disassembly (Stage 2) and complete structure dissolution (Stage 3). MLC, PAK1, and cofilin phosphorylation at Stage 0 is low. At Stage 1 elongation and maturation occur and a PAK1-independent increased in phospho-MLC occurs and acts to balance protrusive and retractile tensions stabilizing invadopodia. PAK1 activity is increased in Stage 1 and PAK1 phosphorylates cofilin to regulate actin assembly. Stage 2, PAK1-dependent phosphorylation of further increases phospho-MLC levels causing a shift in protrusive and retractile tensions dissolving invadopodia. Increased PAK1-mediated cofilin phosphorylation blocks cofilin activity facilitating the disassembly of invadopodia

Since we found that PAK1 regulates cofilin and MLC phosphorylation during invadopodia disassembly, we examined how PAK1 was impacting their phosphorylation status directly within invadopodia during disassembly (T2). Phosphorylated MLC and cofilin were both found to localize at active invadopodia, as indicated by actin cores overlaying areas of degradation (white arrows) and at disassembled invadopodia, as indicated by degradation spots without actin cores (blue arrows) (Fig. 3a, b). Quantification of the composition of invadopodia at T2 in control and PAK1 knockdown cells was performed to assess the number of active invadopodia and the extent to which phosphorylated MLC and cofilin colocalized with active or disassembled invadopodia. Consistent with our previous analysis, 50–60% of PAK1 knockdown cells continued to exhibit actin cores at sites of degradation, compared to 20–30% of control cells, underscoring the role of PAK1 in actin turnover and invadopodia disassembly (Fig. 3c, d). Phospho-cofilin was localized at approximately 20% of invadopodial degradation sites in control cells and a significant reduction was found in PAK1 knockdown cells, with only 1.8 ± 1.9% of degradation sites and 2.1 ± 2.4% of degradation sites with actin cores containing phospho-cofilin (*P* = 0.0016 and *P* = 0.0028, respectively) (Fig. 3c). Similar results were found for phospho-MLC, with approximately 15% of invadopodial degradation sites containing phospho-MLC, which was significantly reduced in PAK1 knockdown cells, with only 2.8 ± 3.9% of degradation sites and 1.8 ± 2.1% of degradation sites with actin cores containing phospho-MLC (*P* = 0.041 and *P* = 0.010), respectively (Fig. 3d). These results demonstrate that PAK1 plays a key role in mediating the phosphorylation of both cofilin and MLC at invadopodia, implicating PAK1 as a key regulator of invadopodia disassembly and retraction. As we found that MLC phosphorylation was increased during invadopodia assembly and disassembly we assessed the effects of impairing MLC activity using a MLC phospho-mutant (MLC2 T18A/S19A) [31]. The percent of control cells forming invadopodia was found to be significantly higher (27 ± 3%) compared to cells stably expressing MLC2 T18A/S19A (9.7 ± 2.6) (Fig. 3e). Taken together this suggests that MLC2 functions in both the assembly and disassembly of invadopodia but only its role in disassembly is mediated by PAK1. Figure 3f proposes a model whereby PAK1-mediated phosphorylation events regulate cofilin and MLC to promote invadopodia disassembly.

### PAK1 regulates invadopodia retraction in vivo

**Fig. 4:**
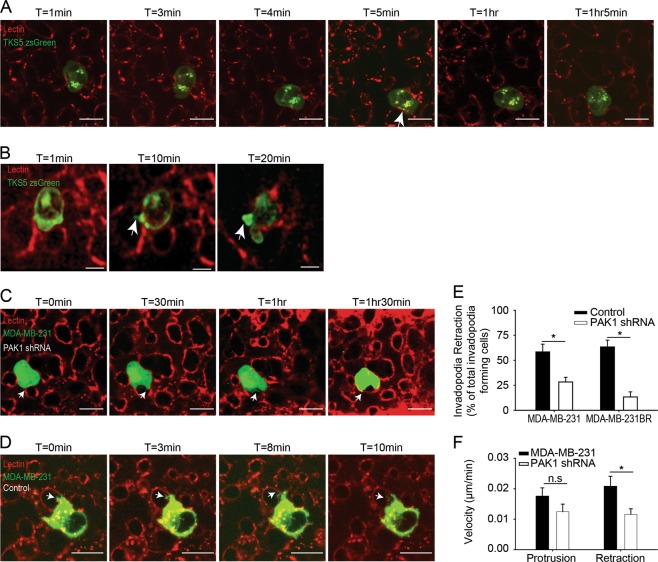
PAK1 mediates in vivo invadopodia retraction. **a** MDA-MB-231 cells transiently transfected with TKS-zsGreen were intravenously injected into the CAM. 2 h post-injection lectin-rhodamine was intravenously injected into the CAM to label the luminal surface of endothelial cells and junctions (red). Cells were imaged in the capillary bed using confocal microscopy. Cell migration is represented in the first three images (T = 1 to T = 4 min), followed by cell arrest and invadopodia formation (T = 5 min), and migration (T = 1hr5min). At T = 5 min invadopodia are visualized by accumulation of TKS5-zsGreen at the luminal surface of endothelial cell (red). White arrow points to invadopodia protruding into the endothelium. **b** Invadopodia protrusion is visualized by TKS5-zsGreen protrusion (white arrow) extending through the endothelial layer (red) (T = 10 min) and extends further (T = 20 min) as the cell invades through the endothelium (red) into the stromal space (black). **c** Representative image of invadopodia formation and extravasation in PAK1 shRNA MDA-MB-231 cells. Cell arrests at the endothelial surface (T = 0), forms protrusions (T = 30 min to T = 1 h) and fully extravasates (T = 1 h 30 min). **d** Representative image of MDA-MB-231 control cell forming and retracting invadopodia protrusion. White arrow points to an invadopodia protrusion (T = 0) which retracts (T = 3 and T = 8 min) followed by cell movement away from the endothelium (T = 10). **e** Quantification of the percentage of MDA-MB-231 and -231BR control and PAK1 shRNA invadopodia retraction in vivo. Invadopodia-forming cells were imaged and the percentage of cells retracting protrusions is shown. **f** Quantification of the velocity of MDA-MB-231 control and PAK1 shRNA cell protrusion and retraction in vivo. Student’s *t*-test was used to determine significant differences. Asterisk denotes a value significantly different from control cells (*p* < 0.05). Scale bar = 10 µm

Given the role of PAK1 in regulating invadopodia retraction in vitro, an in vivo study of invadopodia retraction was performed using the chick chorioallantoic membrane (CAM) model to visualize invadopodia retraction with intravital imaging [2]. We have previously determined using this model that invadopodia are key mediators of extravasation and that impairment of invadopodia formation inhibits cancer cell extravasation [4, 32]. Tks5-zsgreen expressing cells were intravenously injected into the chick CAM where they arrest in the capillary microvasculature, adhere to the endothelium, migrate and form invadopodia-based protrusions, rich in Tks5, that breach the endothelium to mediate extravasation (Fig. 4a, b, Supplemental Movie 1). Cells arrested within the vasculature form invadopodia-based protrusions that breach the endothelial barrier to mediate successful extravasation (Fig. 4b, c), and in some instances these protrusions also retract (Fig. 4d, Supplemental Movie 2). Next, two breast cancer cell lines, MDA-MB-231 zsGreen and MDA-MB-231BR-zsGreen, were injected into the CAM and rates of invadopodia formation (as shown in Fig. 4b) and retraction were assessed. Quantification of retraction events found that 58.3 ± 7.8% of MDA-MB-231 and 63.3 ± 6.8% of MDA-MB-231BR invadopodia-forming cells retracted these protrusions compared to a retraction rate of 28.3 ± 4.6% and 13.3 ± 5.1% in PAK1 knockdown cells (Fig. 4e) (*P* = 0.007 and *P* = 0.010, respectively). The rate at which cell protrusions formed and retracted was also measured in MDA-MB-231 cells and PAK1 knockdown cells. The mean velocity of protrusion was not found to be significantly different between control and PAK1 knockdown cells; however, the rate at which cells retracted their protrusions was found to be significantly slower in knockdown cells (*P* = 0.0018) (Fig. 4f, Supplemental Movie 3).

### Extravasation and metastatic colony formation are mediated by PAK1

**Fig. 5:**
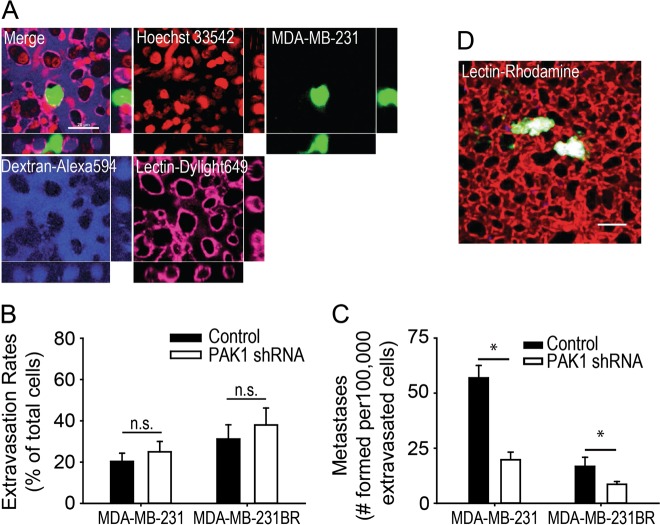
Cell extravasation and metastatic colony formation in the CAM as mediated by PAK1. **a** Intravital imaging of a MDA-MB-231 cell (green) fully extravasated into the extravascular stroma. Endothelial cells were labeled with dylight-649 lectin (violet), nuclei were stained with Hoechst (pseudo-colored red) and rhodamine dextran (pseudo-colored blue) shows the capillary bed. XY and XZ stack views show the MDA-MB-231 cell residing outside the vasculature wrapped around an endothelia cell (violet) in the stromal space. **b** Quantification of MDA-MB-231 and -231BR control and PAK1 shRNA extravasation rates. Cells were i.v. injected into the CAM, cells were counted at time 0 and 24 h post-injection. The percentage of extravasated cells at 24 h is represented as a percentage of total at time 0. **c** Quantification of metastatic colonies 5 days post-injection. Metastatic colonies were visualized and counted in the CAM via fluorescent microscopy. **d** Metastatic MDA-MB-231 colonies (Green) in the CAM as imaged by confocal microscopy. Scale bar = 20 µm. Student’s *t*-test was used to determine significant differences. Asterisk denotes a value significantly different from control cells (*p* < 0.05)

Our data demonstrate that PAK1 regulates invadopodia disassembly in vitro and in vivo. To assess the effects of impaired invadopodia disassembly in vivo, we performed cancer cell extravasation assays, a key step of metastasis, in the CAM model [2, 33]. In this pre-clinical model, PAK1 knockdown did not affect extravasation rates in MDA-MB-231 or MDA-MB-231BR cells (Fig. 5a, b). Similar results were also observed in both the 21MT-1 and 786-O cell lines (Supplemental Fig. 5). Despite similar extravasation rates, MDA-MB-231 and MDA-MB-231BR cells PAK1 knockdown cells formed less metastases relative to the number of extravasated cells, with a significant reduction in colony formation rates observed in PAK1 knockdown cells (Fig. 5c, d and Supplemental Movie 4).

**Fig. 6:**
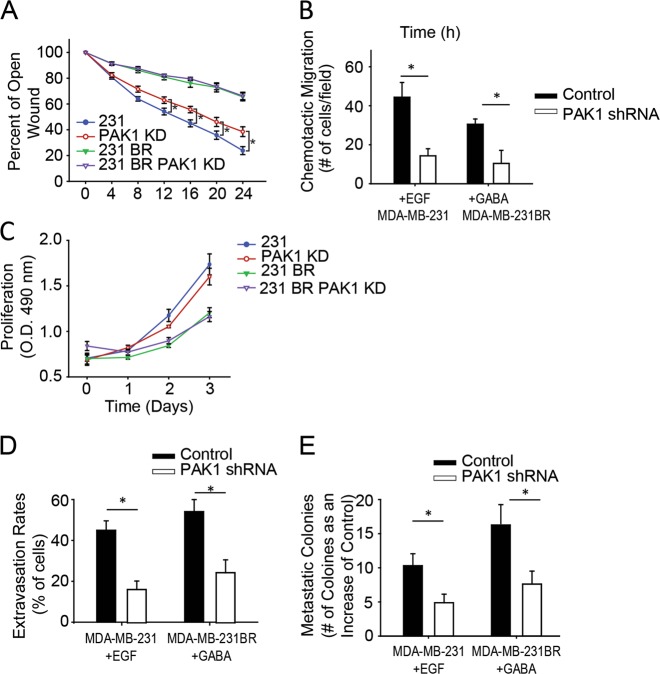
Chemotactic extravasation and metastatic colony formation are regulated by PAK1. **a** Scratch wound migration assay. Cells were plated and grown to confluency. A wound was manually made and cell motility was monitored every 4 h. **b** Chemotactic cell migration. Cells were serum starved and subjected to transwell migration towards 25 ng/ml EGF or 50 µM GABA for 6 h. Cells that migrated to the underside of the membrane were counted and are represented as a percent of uninfected MDA-MB-231BR cell migration. **c** Cells were plated in a 96 well plate and proliferation was measured using a MTT proliferation assay for 3 consecutive days. **d**, **e** 50 ng/ml EGF or 100 µM GABA and control (water) in Matrigel were added on top of the CAM. MDA-MB-231 and MDA-MB-231BR control or PAK1 shRNA cells were i.v injected into the CAM vasculature and **d** counted at the location under the chemoattractant, or control Matrigel, re-counted 24 h later and is represented as a percentage of total cells at time zero. **e** Quantification of metastatic colonies 5 days post-injection. Metastatic colonies were visualized and counted in the CAM via fluorescent microscopy (**f**)

The reduction in metastatic colony formation seen in the MDA-MB-231 and MDA-MB-231BR PAK1 knockdown cells could be a result of reduced motility and post-extravasation proliferation. To assess motility, a scratch wound migration assay was performed and no change in the migration of MDA-MB-231BR PAK1 knockdown cells was found, whereas a small but significant reduction in migration in the MDA-MB-231 PAK1 knockdown cells was observed (Fig. 6a). Next, we evaluated the role of PAK1 in chemotactic migration. Using a transwell chemotactic migration assay we found that MDA-MB-231 and MDA-MB-231BR PAK1 knockdown cells were failed to respond to chemotactic stimulation (Fig. 6b). Proliferation was also assessed and no significant differences were found for either cell line (Fig. 6c). These results suggest a role for PAK1 in regulating cell response to chemotactic stimuli.

From this, we hypothesized that PAK1-mediated invadopodia retraction may be regulated by environmental cues to promote extravasation into chemotactic rich areas. Since MDA-MB-231 cells are known to localize the epidermal growth factor receptor to invadopodia which can promote invadopodia formation under in vitro conditions [13, 18], its impact on in vivo extravasation was evaluated in local chemotactic gradients by adding epidermal growth factor in Matrigel onto the top of the CAM to create a chemotactic concentration gradient (Supplemental Fig. 6, Supplemental Movie 5). Extravasation rates for control and PAK1 knockdown cells were assessed in the presence of chemoattraction (Matrigel plus EGF) or control (Matrigel plus water). In the presence of EGF, control cells extravasated at a significantly higher rate (44 ± 5%) compared to PAK1 knockdown cells (18 ± 4%) (Fig. 6d). EGF significantly increased extravasation rates by greater than 40% whereas PAK1 knockdown cells did not respond to chemotactic stimuli and a slight reduction in extravasation rates was observed (comparison of Fig. 6d to Fig. 5b). These results showed a significant increase in chemotactic extravasation in control cells compared to PAK1 knockdown cells (*P* = 0.0048), suggesting impaired chemotaxis due to invadopodia dysfunction. Metastatic colony formation in the presence of EGF was also significantly higher in control cells compared to PAK1 knockdown (*P* = 0.014) (Fig. 6e).

Brain-tropic basal like breast cancers and HER2+ patient tumors overexpress GABA receptors and metabolize GABA to support tumor growth [34]. We considered the possibility that GABA could act as a chemotactic cue localizing breast cancer cells to the brain. To assess chemotactic extravasation in MDA-MB-231BR cells, GABA was applied to the CAM as a chemoattractant. In the presence of GABA, control cells extravasated at a significantly higher rate (56 ± 7%) compared to PAK1 knockdown cells (23 ± 8%) (Fig. 6e). Furthermore, GABA increased extravasation rates by greater than 40% in control cells, while a slight reduction in extravasation was found in PAK1 knockdown cells (*P* = 0.0043) (comparison of Fig. 6d to Fig. 5c). Metastatic colony formation in the presence of GABA was found to be significantly higher in control cells compared to PAK1 knockdown cells (*P* = 0.030) (Fig. 6e).

**Fig. 7:**
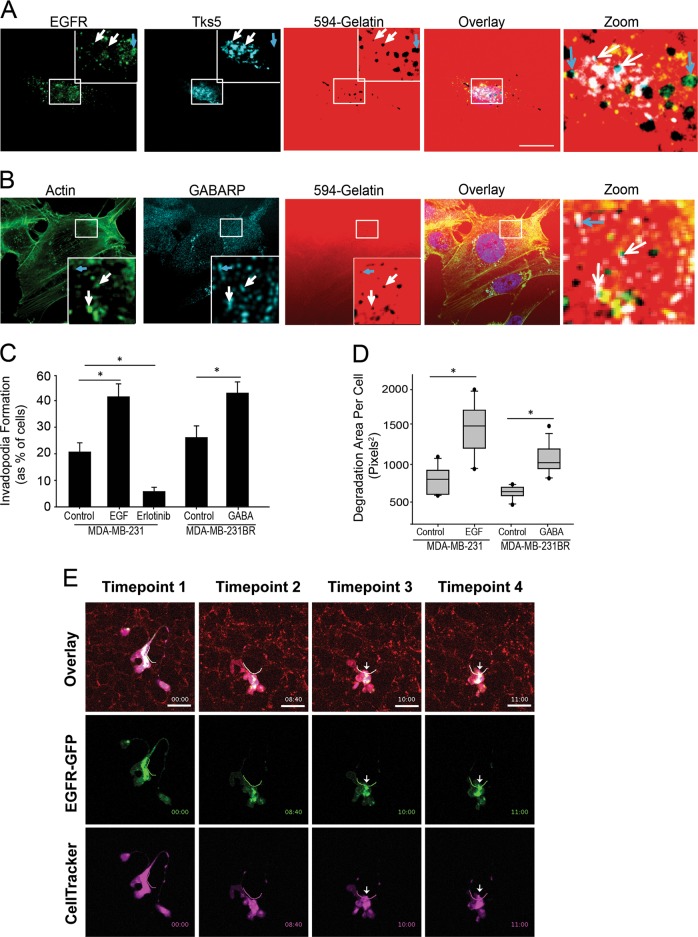
EGF receptor (EGFR) and GABA receptor (GABRP) is localized to invadopodia in vitro and in vivo during cancer cell extravasation. **a** EGFR and **b** GABRP localizes at invadopodia in vitro. Cells were plated on Alexa594-labeled gelatin-coated coverslips for 5 h, fixed, permeabilized and stained using anti-EGFR and anti-Tks5 or anti-GABRP and Alexa488-phalloidin. Single confocal slices of the ventral surface of cells are shown. EGFR (green) and Tks5, and GABRP (cyan) and F-actin colocalization at sites of invadopodium degradation (white arrows) as seen in overlay. EGFR (green) and GABRP (cyan) localize at sites of invadopodium degradation (blue arrows) as seen in overlay. **c**, **d** MDA-MB-231 and MDA-MB-231BR were plated with or without EGF, GABA, or Erlotinib as indicated, fixed, permeabilized, and stained for Alexa488-phalloidin. **c** Percentage of cells forming invadopodia was quantified. **d** Invadopodium-based degradation of the 594-gelatin matrix of individual cells was quantified using ImageJ software. Means ± SEM from 3 independent experiments in which 10–20 cells per sample were measured are shown. Mean ± S.E. (error bars) from three independent experiments. Student’s *t*-test was used to determine significant differences. Asterisk denotes a value significantly different from control cells (*p* < 0.05). Scale bar = 10 µm. **e** MDA-MB-231 cells were transiently transfected with a vector to express EGFR-GFP protein. Cells were i.v. injected into the chorioallantoic membrane and intravital imaging was performed to visualize EGFR-GFP compartmentalization to invadopodia during cancer cell extravasation. Arrows point to invadopodia breaching the endothelium of the CAM that appear to localize EGFR-GFP during cancer cell extravasation. EGFR-GFP rich invadopodia are most prominent in the last timepoint (#4)

The pi subunit of the GABA receptor (GABRP) promotes cellular protrusions and migration and is upregulated in basal-like primary breast tumors that result in brain metastases [35]. GABRP localization in MDA-MB-231BR cells revealed localization at invadopodia in vitro (Fig. 7a). In MDA-MB-231 cells EGFR was localized to invadopodia (Fig. 7b). To determine the effect of EGF and GABA on invadopodia formation EGF or GABA was added to MDA-MB-231 and MDA-MB-231BR cells, respectively. The percentage of cells forming invadopodia was quantified and a significant increase in invadopodia was found with GABA and EGF treatment (Fig. 7c). Impairing EGFR signaling using Erlotinib significantly reduced the percentage of cell forming invadopodia (Fig. 7c). In addition, invadopodia-based degradation was quantified and treatment of cells with GABA or EGF significantly increased the amount invadopodia-based degradation (Fig. 7d). This suggests that chemotactic ligands such as EGF and GABA are important for invadopodia function. In vivo, EGFR-GFP was also observed to be localized to the apical portion of the breaching invadopodia during cancer cell extravasation (Fig. 7c and Supplemental Movie 6). This has not been visualized in vivo previously. Taken together, these results demonstrate that PAK1 regulates chemotactic extravasation by dissolving invadopodia in the absence of external cues, and this may facilitate tumor cell extravasation and metastatic colony formation in chemotactic rich areas supportive of growth.

### Impairing PAK1-mediated invadopodia disassembly reduces tumor burden

**Fig. 8:**
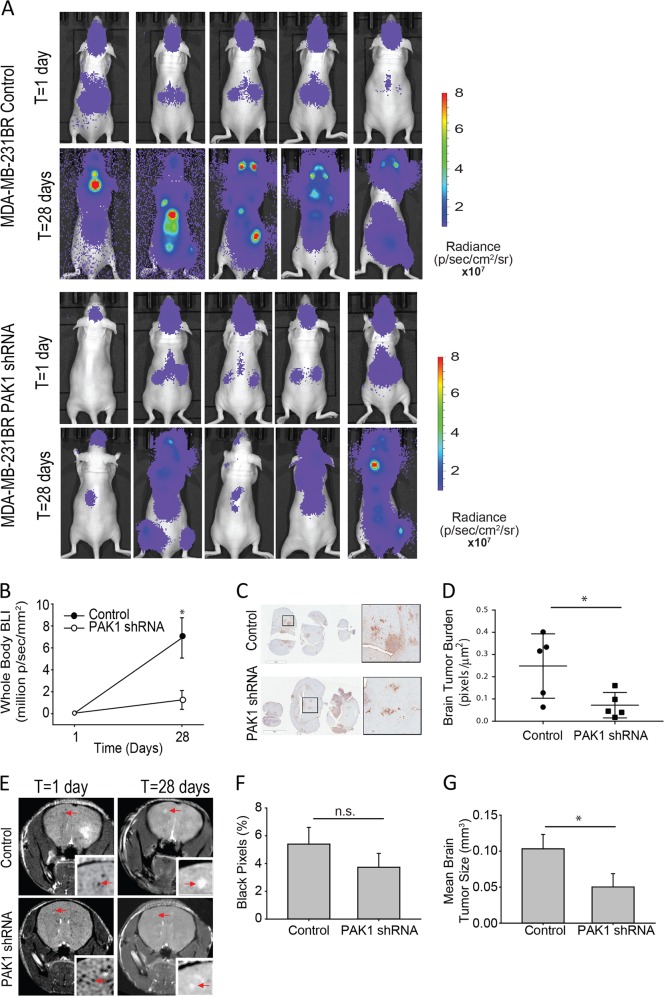
PAK1-mediates brain metastases in a mouse model. MDA-MB-231BR control or PAK1 shRNA expressing firefly luciferase were iron loaded followed by intracardiac injection into nude mice. **a** Images of bioluminescence imaging (BLI) and MRI imaging at day 1 and day 28 post-injection. Cells and tumors were also detected using BLI at day 1 and on day 28. Correlative H&E-stained sections of the area indicated show morphology of tumors. **b** BLI signal as measured at day 1 and at end-point (day 28). **c**, **d** Histological staining of brain sections using vimentin are represented (**c**) and tumor burden based on DAB-positive pixels was quantified (**d**). **e** Representative MR images at day 1 and day 28 post-injection. MR images show signal voids representing single cells residing in the brain at day 1, and at day 28 brain metastases appear as a region of hyperintensity on MRI images (**f**) Tumor voids were counted on day 1 from MR images and are represented as a percentage of black pixels. **g** Individual tumor volumes were calculated from MR images and the mean volume per tumor is represented. Student’s *t*-test was used to determine significant differences. Mean ± S.E. (error bars) is represented

To further assess the role of PAK1-mediated invadopodia disassembly in metastasis, the ability of MDA-MB-231BR PAK1 knockdown cells to form metastatic tumors was evaluated in an experimental metastasis mouse model [36]. MDA-MB-231BR cells are a highly metastatic brain homing cell line which readily form brain metastases. MDA-MB-231BR control and PAK1 knockdown cells stably expressing Firefly luciferase were loaded with iron oxide nanoparticles prior to ultrasound-guided intracardiac injection into mice. Multimodality imaging was performed to confirm successful injection and to quantify cell arrest and endpoint measurements of total tumor burden and viability [37]. Mice were imaged by bioluminescence imaging (BLI) and magnetic resonance imaging (MRI) post-injection on Day 1 and at endpoint on Day 28. BLI confirmed successful injection of MDA-MB-231BR cells and arrest of the brain-tropic cells in the brain (Fig. 8a). BLI signal on Day 1 was not found to be significantly different between control (5.3 × 10^3^ ± 1.1 radiance) and knockdown (5.5 × 10^3^ ± 1.7 radiance).

We then quantified tumor burden at endpoint, Day 28, using BLI signal (Fig. 8a, b) and found a significant reduction in total tumor burden in mice injected with PAK1 knockdown cells (1.2 × 10^5^ ± 0.8 radiance) compared to control cells (6.9 × 10^5^ ± 1.8 radiance) (*p* = 0.02) (Fig. 8e). Luciferase expression between control (1.0 × 10^5^ relative light units per µg protein) and PAK1 knockdown (1.2 × 10^5^ relative light units per µg protein) cell lines was determined by firefly luciferase assay and no difference in expression were found. Presence of tumor(s) was also confirmed using histology (Fig. 8c). Histological quantification of brain tumor burden was performed and a significant reduction in tumor burden was found in mice with PAK1 knockdown cells compared to control (*p* = 0.04) (Fig. 8d).

MRI demonstrated similar results showing signal voids within the brain for both control and PAK1 knockdown cells at Day 1 (24 h post-injection) and quantification of the cell voids in the brain on Day 1 showed no significant difference between control (5.4 ± 1.2% black pixels) and PAK1 knockdown (3.73 ± 1.0% black pixels) (Fig. 8e and F-left column). Additionally, we performed MR image analysis at day 28 to quantify tumor size. MRI at day 28 showed tumor growth at the sites where cells resided at T = 1 day (Fig. 8e: right column, red arrows) as regions of signal hyperintensity. Using MR image analysis at day 28, brain tumor size was quantified and it was found that brain tumors present in mice injected with PAK1 knockdown cells were significantly smaller (0.05 ± 0.01 mm^3^) than control tumors (0.10 ± 0.02 mm^3^) (*p* = 0.015) (Fig. 8g). Overall, these results suggest that proper invadopodia dynamics regulated by PAK1 is required for efficient invasion and metastatic tumor growth.

## Discussion

We report that invadopodia dynamics are regulated by PAK1 in response to chemotactic ligands to mediate effective metastatic outgrowth, particularly in the brain. PAK1 can mediate actin dynamics through cortactin [23] and p27^Kip1^ [24], and here we demonstrate that invadopodia disassembly is regulated by phosphorylation of cofilin and myosin light chain by PAK1. PAK1 regulates cofilin and MLC phosphorylation at sites of invadopodium degradation during disassembly, which has functional consequences during cancer cell extravasation, a key step of the metastatic cascade [32]. Active cofilin is known to sever actin filaments to produce free barbed ends that induce actin polymerization and cause enlargement of cell protrusions [11], and this activity is blocked by phosphorylation of Serine3 on cofilin [27]. It is well established that PAK1 regulates cofilin by directly phosphorylating LIM kinase thereby activating it and causing downstream phosphorylation of cofilin [27]. When PAK1 activity is blocked, cofilin phosphorylation is subsequently impaired and larger static invadopodium actin cores are formed in vitro, suggesting that enhanced cofilin activity drives excessive actin polymerization leading to increasing actin core size. Enlarged static invadopodia as a result of PAK1 impairment may explain the slight increase in extravasation rates observed in PAK1 knockdown cells.

F-actin polymerization is essential for invadopodia protrusion, while tensile forces generated by both F-actin and myosin facilitate contractility. Myosin light chain activity, via its phosphorylation, is not necessary for myosin-actin binding but is required to generate contractility. PAK1 regulation of myosin light chain (MLC) phosphorylation has been documented under different conditions to either block [27, 38, 39] or promote MLC phosphorylation, thus regulating its activity [29, 40, 41, 42]. MLC regulation of contractile force has been examined in podosomes showing that low MLC activity renders podosomes static and non-protrusive, whereas increased activity causes podosome disassembly [43]. Our results suggest that a similar dynamic is present at invadopodia. At T = 0 min when invadopodia protrusion is limited by the matrix metalloproteinase inhibitor GM6001, low levels of phospho-MLC were observed, hence representing a static state. Phospho-MLC levels significantly increased during maturation and again during disassembly. We posit that invadopodia are static when levels of phospho-MLC are low, elongate and mature with increased phospho-MLC by balancing protrusive and retractile tensions, and then dissolve upon enhanced MLC phosphorylation. While we found that PAK1 activity positively regulated MLC phosphorylation during disassembly, it did not have an effect on MLC phosphorylation levels during maturation. The mechanisms by which PAK1 is able to regulate an oscillating MLC phosphorylation state during invadopodia assembly and disassembly needs to be further explored. In addition, the observation that PAK1 did not alter MLC activity during maturation suggests alternative pathways may also be involved in MLC regulation at invadopodia.

Invasion into stroma is a hallmark feature of metastatic cancer cells, but the environmental cues and cellular machinery responsible for coordinating the invasive phenotype were unclear until now. Our investigation into the role of invadopodia dynamics has provided the first in vivo evidence of the functional consequences of invadopodia retraction. Our intravital imaging findings revealed a chemosensing apparatus utilized by invadopodia during extravasation, a key step of metastasis. PAK1 depletion in metastatic cancer cells did not impact invadopodia formation but significantly impaired invadopodia disassembly in vivo. Under conditions of no chemotactic stimuli, the inability to retract invadopodia did not impair extravasation rates. However, under conditions of abundant chemotactic stimuli such as EGF and GABA, metastatic cancer cells demonstrated significantly increased extravasation rates, while PAK1 knockdown cell types did not respond to stimuli leaving extravasation rates unaltered. The chemosensory ability of invadopodia is pronounced in the presence of chemotaxis stimuli and may be responsible for guided cancer cell extravasation into specific microenvironments permissive of metastatic colony growth. Hence, PAK1 is responsible for invadopodia-based chemotactic invasion and this finding is corroborated by previous reports describing PAK1 mediated chemotaxis in macrophages [28].

Cancer cell extravasation into any microenvironment at a distant site will be co-dependent on the tumor cell and “fertility” of the soil. Our observations regarding invadopodia and chemotaxis reconcile various reports describing the efficiency or lack thereof of cancer cell extravasation at various sites [34, 44–48]. Our in vitro and chick CAM assays demonstrate that invadopodia are chemosensing protrusions that trigger extravasation into microenvironments rich in factors that permit cancer cell growth, and that PAK1 is central to this process by inhibiting extravasation into stroma that is not optimal for metastatic colonization. This is consistent with our results using a mouse model of experimental metastasis that shows that PAK1 knockdown results in significantly smaller brain metastases compared to controls, highlighting that PAK1 plays an important role in metastatic outgrowth post-extravasation. In summary, PAK1 is responsible for guided cancer cell extravasation because invadopodia are able to respond to specific stimuli before prompting the cell to undergo transendothelial migration. PAK1 inhibition is therefore a relevant option for antagonizing metastatic dissemination.

## Materials and methods

### Reagents and constructs

All chemicals were purchased from Sigma-Aldrich (St. Louis, MO) or Fisher Scientific (Nepean, ON, Canada) unless otherwise indicated. The PAK1 inhibitor IPA-3 was purchased from EMD Chemicals (Gibbstown, NJ). GABA was purchased from Abcam (Toronto, ON). Antibodies to the following proteins were obtained from the indicated suppliers: Anti-TKS5 (SH3 #1) EMD Millipore 09-403, Myosin Light Chain 2 ABM Y021157, Myosin Light Chain 2 antibody [EPR3741] Abcam ab92721, p-MYL9 Antibody (Thr 18/Ser 19)-R Santa Cruz Biotechnology sc-12896-R, Phospho-PAK1 (Thr423)/PAK2 (Thr402) Antibody Cell Signaling Technology 2601S, Anti-PAK1 antibody Abcam ab40852, Cofilin (phospho S3) antibody Abcam ab12866, Anti-Cofilin antibody (ab42824) Abcam ab42824. All secondary antibodies and Alexa Fluor 647–labeled phalloidin were purchased from Invitrogen (Burlington, Canada). PAK1 shRNA construct (CCAAGAAAGAGCTGATTATT) and control plasmid pLKO.1 was kindly provided by Dr. Jason Moffat and the Ontario Institute for Cancer Research (OICR) Genomics Facility. The pEGFR-GFP plasmid was obtained from Addgene (Plasmid #32751). The MLC2 T18A,S18A plasmid was obtained from Addgene (Plasmid # 35681).

### Cell lines

MDA-MB-231 cells (ATCC HTB-26) were grown in DMEM supplemented with 10%FBS and 786-0 cells (ATCC CRL-1932) were grown in RPMI supplemented with 10%FBS. MDA-MB-231BR (BR-brain derived) cells stabling expressing firefly luciferase were a kind gift from Drs Patrica Steeg and Brunilde Gril. These cells were grown in DMEM supplemented with 10%FBS as described [49]. The 21MT-1 cell line was obtained as a kind gift of Dr. Vimla Band [50]. These cells were maintained in culture in AMEM supplemented with 10% fetal bovine serum (FBS), 2 mM l-glutamine (both from Gibco Life Technologies, Grand Island, NY, USA), insulin (1 ug/ml), epidermal growth factor (12.5 ng/ml), hydrocortisone (2.8 mM), 10 mM 4-(2-hydroxyethyl)-1-piperazineethanesulfonic acid (HEPES), 1 mM sodium pyruvate, 0.1 mM non-essential amino acids and 50 ug/mL gentamycin reagent (all from Sigma Chemical, St Louis, MO, USA), as described [51].

### The ex ovo chick embryo model

Extravasation assays were performed as previously described [2]. Briefly, stable cell lines generated by lentiviral infection of control or PAK1 shRNA were lifted in 0.5% trypsin with 0.05 mM EDTA, pelleted and washed three times with PBS. Cells were counted and resuspended at 1 × 10^6^ cells/ml. 100ul of cells was intravenously injected into the CAM using a disposable micropipette syringe as described previously using a day 13 chicken embryo and analyzed for extravasation 24 h post-injection or metastases at 5 days post-injection using an wide field epi-fluorescence microscope. Chemosensing assays were performed by diluting either EGF (50 ng/ml), GABA (100 µM), or control (water) in Matrigel. For extravasation, a 10 ul drop of either control and EGF or GABA was placed on the CAM prior to injection of cells. Cells were counted directly under the matrigel drop area post-injection and 24 h later. For metastatic colony formation, a quarter of the CAM was covered with a thin layer (100 ul) of control and either EGF or GABA and colonies were counted in these areas 5 days post-injection. For invadopodia formation/retraction, cells were imaged 4 h post-injection and cells forming invadopodia were monitored over two hours to assess retraction. To image cells and colonies in the CAM 50 ul Lectin-Rhodamine/Dylight 649 lectin (1:10 in PBS) and Hoechst (1:300 in PBS) was intravenously injected into the CAM and analyzed using a Nikon upright confocal microscope. (12–16 chicken embryos were injected for each construct; *n* = 3).

### Proliferation assay

Cells were seeded in triplicate in 96 well cell culture plates (5000 cells/well) in DMEM media containing 10% FBS. Immediately after seeding, or at the indicated day intervals, 20 ul of Celltiter96® Aqueous One Solution (Promega) was added in triplicate to each sample and incubated for 2 h. After incubation, the OD at 490 nm was measured using a Bio-Rad microplate reader to quantify changes in cell proliferation over the indicated time period.

### Scratch closure assay

Cells were seeded in 6-well cell culture dishes (1 × 10^6^ cells per well) in DMEM media containing 10% FBS and incubated overnight to form a monolayer. A scratch in the cell monolayer was then created using a 100 uL pipette tip, and cell debris was subsequently removed by washing the cells 3X with PBS. Cells were then incubated in the environmental chamber (37 ^o^C, 5% CO_2_) of an EVOS FL Auto Cell Imaging System (Thermo Fisher), and imaged at the same location of the monolayer scratch every 5 min for 24 h. Images were then compiled into timelapse videos using ImageJ software. To quantify scratch closure as a measure of migration, the width of the scratch for each sample was measured every 4 h for 24 h, and normalized to the initial (0 h) width of the scratch. Scratch closure is presented as a mean percentage of the initial scratch ± SEM.

### In vivo protrusion analysis

Cells expressing fluorescent ZsGreen protein were injected intravenously into day 13 avian embryos (1 × 10^5^ cells in 100 ul PBS). Approximately 3 h after cell injection, embryos were injected intravenously with fluorescent DyLight 649 labelled Lens Culinaris Agglutin (Vector labs) to label the vasculature of the embryos. Following lectin injection, embryos were imaged using a Nikon A1R+ confocal microscope (1.2 au) with a 60x oil-immersion lens. Z-stacks were acquired (0.5 µm step size) with the resonant scanner every 30 sec for 30 min to capture the embryos vasculature and associated extravasating cells during that time period. The acquired Z-stacks were then compiled into a max-intensity projection using ImageJ software to create timelapse videos. To examine the dynamics of cell protrusions in vivo during extravasation, the ZsGreen cell channel was analyzed using the protrusion analysis feature of the ADAPT plugin for ImageJ [52]. The mean protrusion and retraction velocity (µm/min) at each time point for each cell analyzed (*n* = 5 per group) was compiled and averaged to reflect the protrusive dynamics of the different cell types in vivo, and displayed as mean velocity ± SEM. For experiments in which EGFR-GFP protein localization to invadopodia was performed, MDA-MB-231 cells were transiently transfected and then after 24 h post-transfection, 100,000 cells/embryo were injected and at T = 4 h post-injection, 75 µL of 10X diluted DyLight A649 lectin was injected. Intravital imaging was performed as previously described above.

### Migration assays

Boyden Transwell migration chambers, 8 µm (Costar) were left uncoated and used to assess chemotactic migration. Cells from stable lines, or uninfected controls, were serum starved for 8–20 h, lifted, counted and 50,000 cells per well, in serum free media, were added to the top of the transwell chamber. Cells were serum-starved for an additional 3 h and then DMEM/EGF (25 ng/ml) or GABA (50 µM) was added to the bottom well and cells were allowed to migrate for 12 h. The bottom of the membrane was fixed in 4% PFA, stained with DAPI and mounted on coverslips. 10 fields of cells on the membrane were counted, per experiment, using fluorescence microscopy. The data are presented as the number of MDA-MB-231 cells that migrated to the bottom membrane.

### Invadopodia formation assays

Invadopodia formation assays were performed as previously described [53]. Briefly, coverslips were coated with 50 µg/ml poly-l-lysine (Sigma), followed by 0.5% gluteraldehyde (sigma) and inverted on an 80 ul drop of unlabeled, Alexa488-labeled,or Alexa594-labled, incubated with 5 mg/ml NaBorohydride (Sigma), and washed extensively with PBS. Cells were serum starved for 6–12 h, lifted and plated on coverslips with 50 um GM6001 for 3 h (T0), followed by extensive washes with PBS, incubated for 1 (T1) or 2 h (T2), as indicated, fixed, permeabilized and stained with indicated antibodies and phalloidin. GABA (1 mM), EGF (100 ng/ml) or Erlotinib (20 µM) was added, as indicated, during cell plating for 4-hrs. For analysis of invadopodia degradation and actin core size samples were imaged using a 63 × (NA 1.4) lens on a Leica DM-IRE2 upright microscope with a Leica TCS SP2 system (Leica, Heidelberg, Germany) and areas of degradation per cell or actin cores were analyzed using ImageJ software Analyze Particle program. For Western blot analysis, cells were plated on unlabeled gelatin and treated as above followed by lysis at T0, T1, and T2.

### BLI procedure

MDA-MB-231BR cells stably expressing luciferase were used to generate knockdown and control cells. Bioluminescent of control and knockdown cells was assessed using a dual luciferase reporter assay and measured on a luminometer to show equivalent expression levels. In vivo BLI was performed on IVIS Lumina XRMS optical/X-ray scanner (PerkinElmer). Mice were anesthetized with isofluorane (2% in 100% oxygen) using a nose cone attached to an activated carbon charcoal filter for passive scavenging. 24 h post-intracardiac injection whole body BLI imaging was used to screen mice. Mice with BLI signal from the brain were then imaged with MRI on day 1 and 28. Mice received 150 μL of D-luciferin (30 mg/mL) intraperitoneally and BLI images were captured every 5 min for up to 35 min. For each image, a standardized full body region of interest was drawn to encompass the whole mouse and the average radiance (photons/sec/cm^2^/sr) was calculated using Living Image software (PerkinElmer, version 4.5.2).

### Magnetic resonance imaging

All images were acquired on a 3.0-T GE Excite MR750 clinical scanner (General Electric, Mississauga, Canada) using a custom-built insertable gradient coil (inner diameter = 17.5 cm, gradient strength = 500 mT/m, and peak slew rate = 3000 T/[m s]) and a custom solenoidal mouse head radiofrequency coil (inner diameter = 1.5 cm). Mice were anesthetized (1.5% isoflurane in oxygen), and temperature was maintained using warm saline bags during the scans. In vivo MR images were acquired using a 3D balanced steady-state free precession (bSSFP) pulse sequence. These images were used to detect signal voids resulting from iron-labeled cells post–cell injection on day 1 and to identify metastases in the whole mouse brain on day 28. The parameters for the bSSFP scans were as follows: resolution = 100 × 100 × 200 μm, repetition time = 8 or 10 milliseconds, echo time = 4 or 5 milliseconds, flip angle = 35°, signal averages = 2, radiofrequency phase cycles = 8, scan time = 28 or 36 min.

MRI images were analyzed using OsiriX software (Pixmeo, SARL, Bernex, Switzerland). The number of dark pixels within the total brain volume was determined from day 1 images; The brain was outlined as a region of interest where a threshold value is set based on the mean value of signal void ± 2 standard deviations. The total number of black pixels under this threshold value was obtained from the entire brain volume signal intensity histogram. For day 28 image data, brain metastases were manually traced by a single observer. 3D tumor volumes were reconstructed using the OsiriX volume algorithm from the manual segmentation of a region of interest around each tumor boundary in every bSSFP image slice for each mouse.

### Animal preparation

For iron labeling, 2 × 10^6^ cells were plated in a 75 cm^3^ flask, supplemented with DMEM containing 10% FBS, and allowed to adhere for 24 h. Then cells were incubated for an additional 24 h with 10 mL media containing 25 μg/mL of MPIO beads (0.9 um in diameter, 63% magnetite, labeled with Flash Red; Bangs Laboratory, Fishers, IN, USA). Cells were washed once in the flask with Hanks balanced salt solution (HBSS) and then trypsinized with 0.25% Trypsin-EDTA. The cells were then collected and thoroughly washed three more times with Hanks balanced salt solution (HBSS) to remove unincorporated MPIO before cell injection.

Female nude mice (nu/nu, aged 6–8 weeks, from Charles River Laboratories, Wilmington, MA) were housed in a pathogen-free barrier facility, and all experiments were approved by the Animal Use Subcommittee of the University Council on Animal Care at the University of Western Ontario. Cells suspended in 0.1 mL of HBSS were delivered to female nude mice, anesthetized with 2% isoflurane in oxygen, by intracardiac injection to the beating left ventricle of the mouse heart using ultrasound guidance. The brain metastatic breast cancer model cell line, MDA-MB-231BR cells stably expressing luciferase and control or shRNA PAK1, were injected intracardially into mice. The control group (*n* = 5) and PAK1 shRNA group (*n* = 5) were injected with 100,000 cells per mouse.

### Histology

At end point, day 28, all mice were sacrificed by pentobarbital overdose. Brains were excised and placed in formalin for at least 24 h. Fixed brains were processed, paraffin embedded, and serially cut into 5-μm sections. Tissue sections were deparaffinized, rehydrated, and histologically stained with hematoxylin and eosin (H&E) or anti-vimentin antibody. Quantification of vimentin staining was performed using Aperio Image Scope software, Pixel Quantification v9. 20- 40 brain sections per mouse were quantified.

### Statistics and study design

All statistical analysis was performed using Sigma Plot. One-way ANOVA followed by Holm-Sidak post hoc tests or Student’s *t*-test analysis was performed on experimental repeats of *n* = 3 or greater. Mouse sample size was determined using an α of 0.05 and 80% power. Chick embryo sample size was determined using an α of 0.05 and 95% power. Mouse studies and chicken embryo studies were performed blinded. No animals were excluded from study results. In each figure the mean is presented with standard error of the mean.

## Supplementary information

Supplementary Figure 1

Supplementary Figure 2

Supplementary Figure 3

Supplementary Figure 4

Supplementary Figure 5

Supplementary Figure 6

Supplemental Movie 1

Supplemental Movie 2A

Supplemental Movie 2B

Supplemental Movie 3A

Supplemental Movie 3B

Supplemental Movie 4

Supplemental Movie 5A

Supplemental Movie 5B

Supplemental Movie 5C

Supplemental Movie 5D

Supplemental Movie 6

Supplemental Movie 7
