# Supplementary figures and images for: Invadopodia are chemosensing protrusions that guide cancer cell extravasation to promote brain tropism in metastasis
Source: Oncogene. 2019 Jan 16;38(19):3598–615. doi: 10.1038/s41388-018-0667-4 (PMC6756237; doi:10.1038/s41388-018-0667-4)

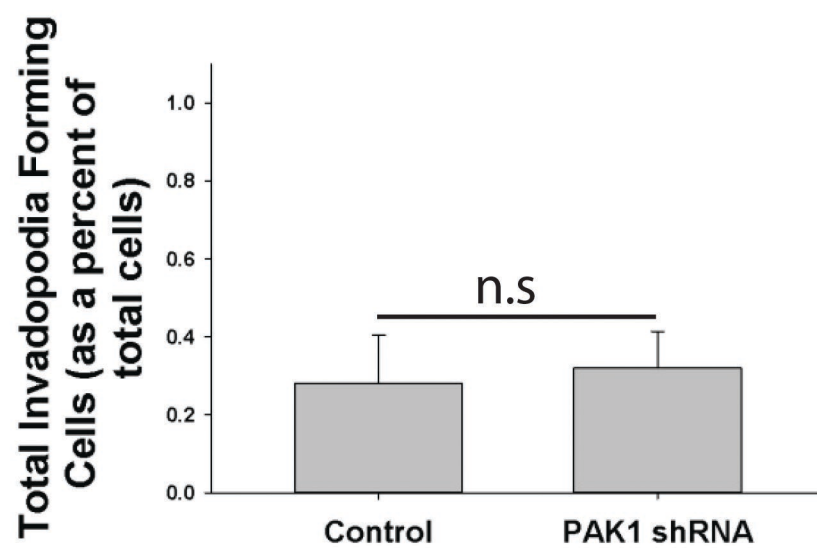

Supplemental Figure 1 Williams *et al.*

Supplement: Supplementary file 1 — Supplementary Figure 1 [file 41388_2018_667_MOESM1_ESM.pdf]

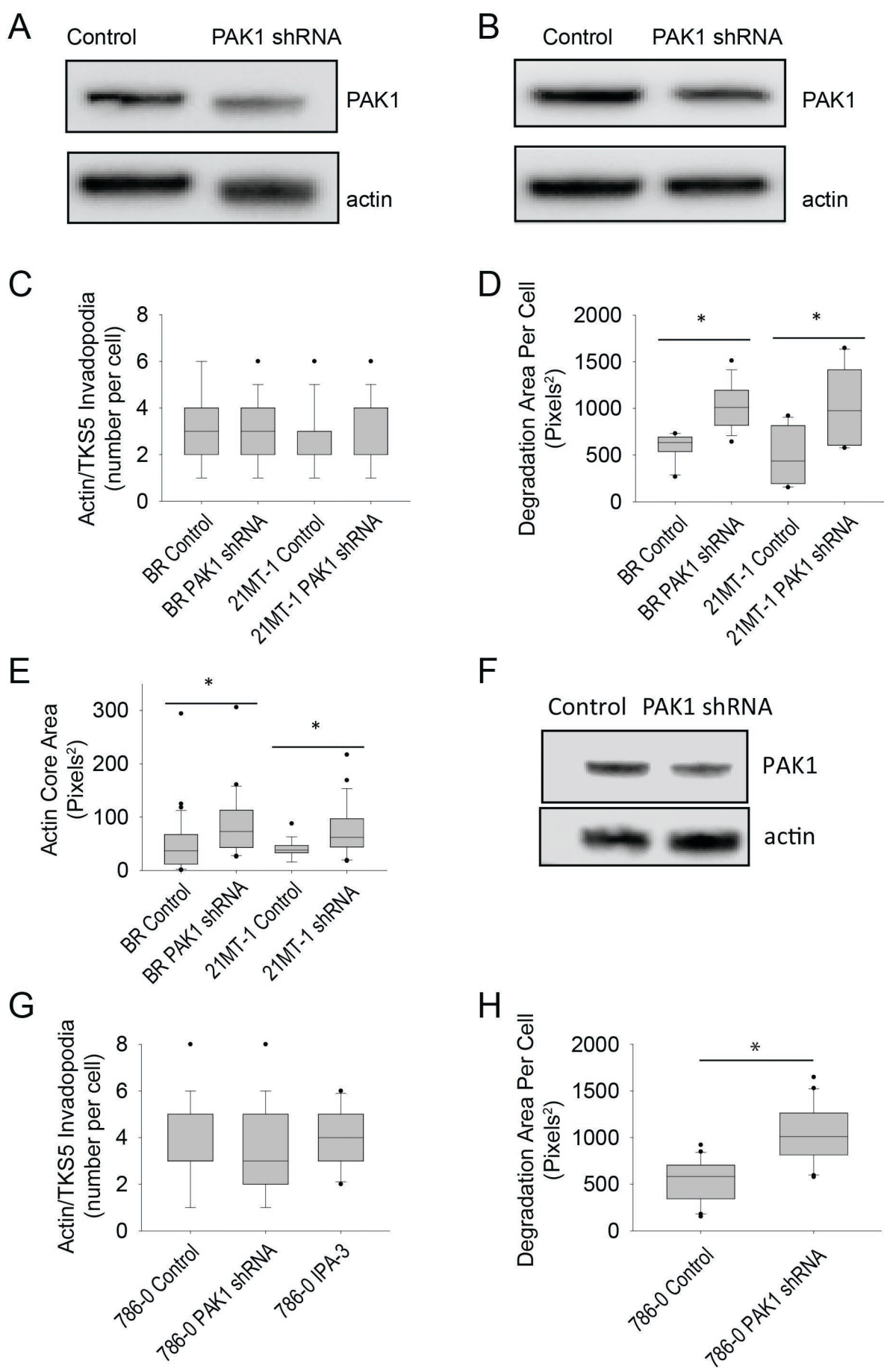

Supplemental Figure 2 Williams *et al.*

Supplement: Supplementary file 2 — Supplementary Figure 2 [file 41388_2018_667_MOESM2_ESM.pdf]

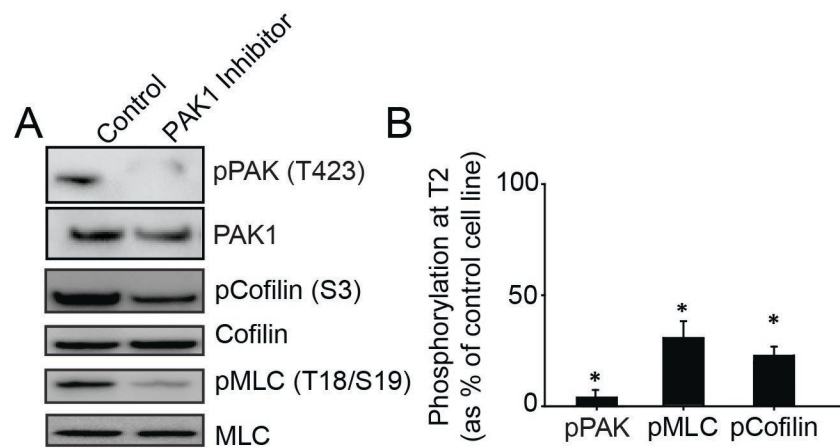

Supplemental Figure 3 Williams *et al.*

Supplement: Supplementary file 3 — Supplementary Figure 3 [file 41388_2018_667_MOESM3_ESM.pdf]

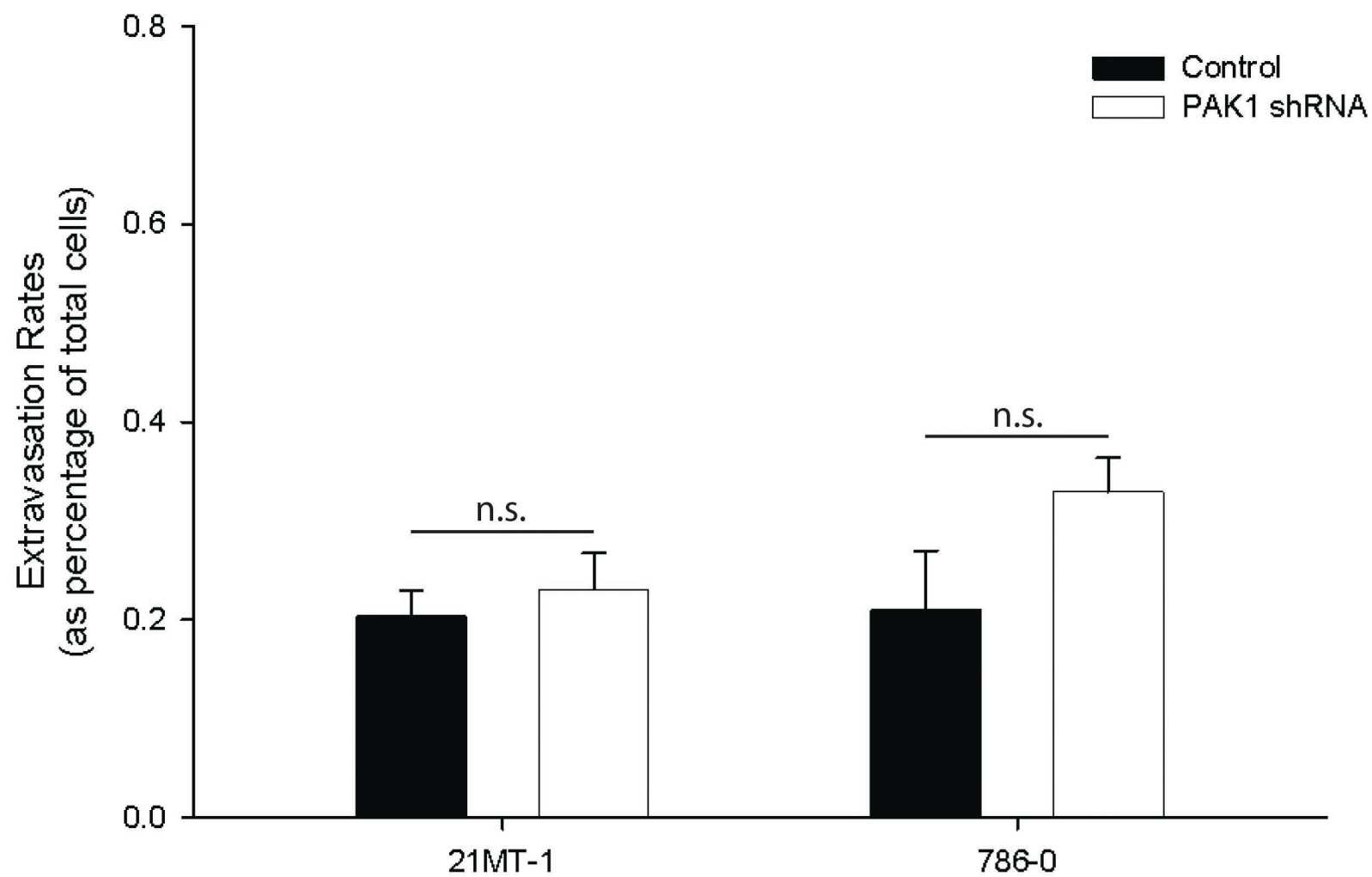

Supplemental Figure 5 Williams *et al.*

Supplement: Supplementary file 5 — Supplementary Figure 5 [file 41388_2018_667_MOESM5_ESM.pdf]

T=3hr

T=6hr

T=9hr

T=12h

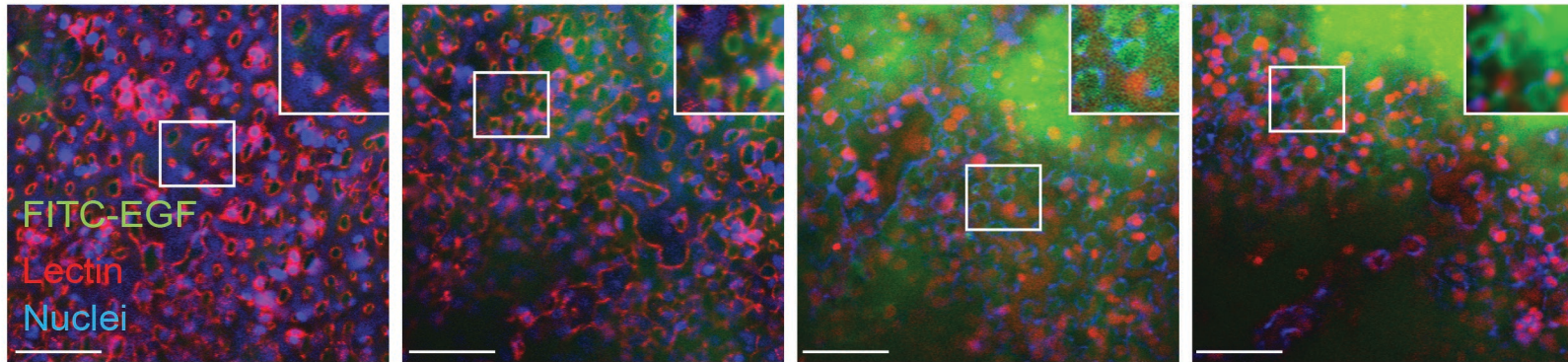

Supplemental Figure 6 Williams *et al.*

Supplement: Supplementary file 6 — Supplementary Figure 6 [file 41388_2018_667_MOESM6_ESM.pdf]
