# Supplementary material for: Invadopodia are chemosensing protrusions that guide cancer cell extravasation to promote brain tropism in metastasis
Source: Oncogene. 2019 Jan 16;38(19):3598–615. doi: 10.1038/s41388-018-0667-4 (PMC6756237; doi:10.1038/s41388-018-0667-4)
Supplement: Supplementary file 4 — Supplementary Figure 4 [file 41388_2018_667_MOESM4_ESM.pdf]

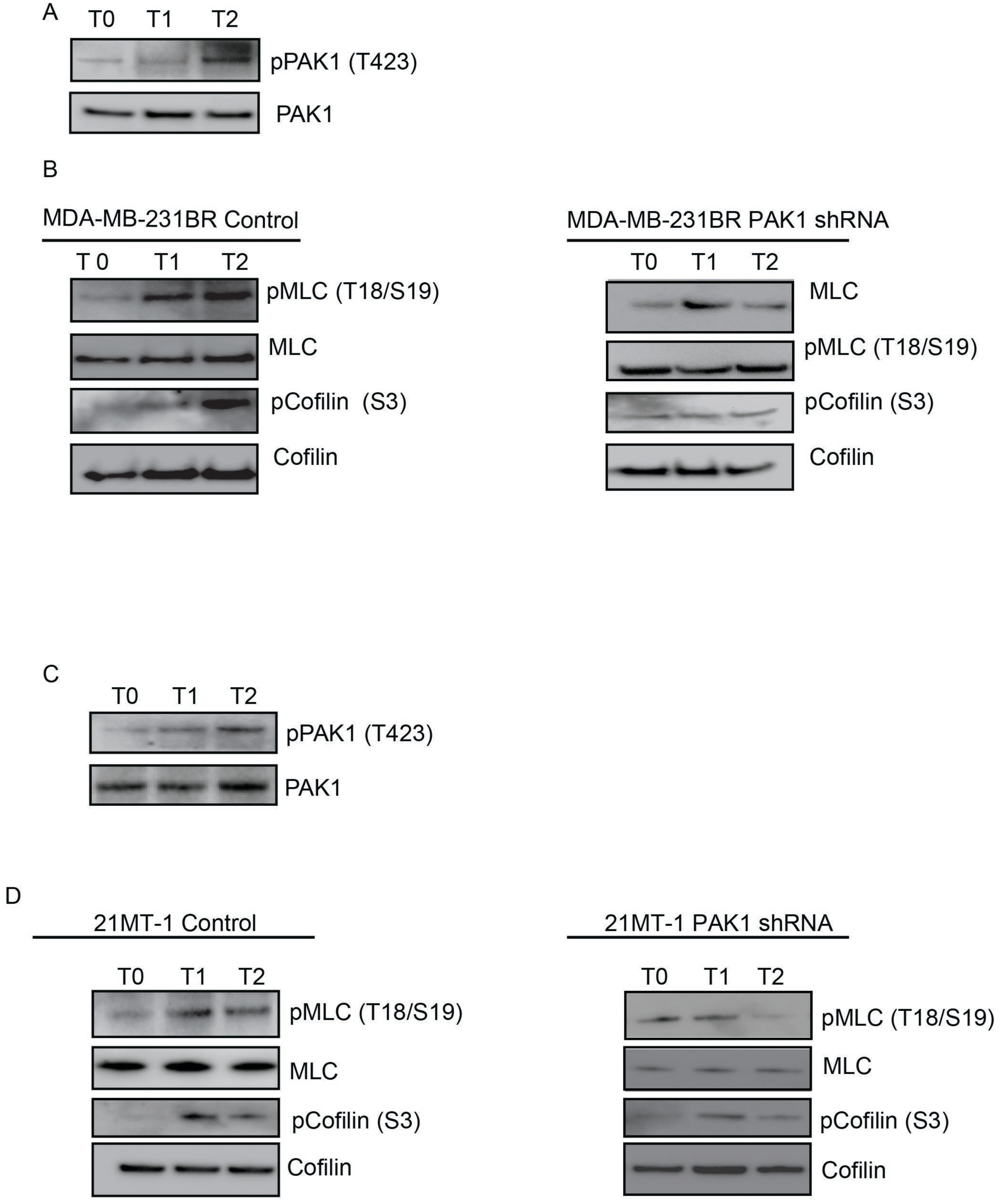

Supplemental Figure 4 Williams *et al.*
