# Supplementary material for: Invadopodia are chemosensing protrusions that guide cancer cell extravasation to promote brain tropism in metastasis
Source: Oncogene. 2019 Jan 16;38(19):3598–615. doi: 10.1038/s41388-018-0667-4 (PMC6756237; doi:10.1038/s41388-018-0667-4)
Supplement: Supplementary file 7 — Supplemental Movie 1 [file 41388_2018_667_MOESM7_ESM.docx]

**Supplemental Movies**

**Movie 1: Cell migration and invadopodia formation in the chick CAM.**

An MDA-MB-231 cell transiently transfected with TKS5-zsGreen within the vasculature of the CAM. Lectin-rhodamine labels the luminal surface of endothelial cells and junctions (red). Cells were imaged in the capillary bed using confocal microscopy. Movie shows migration and arrest of the cell whereby a TKS5-zsGreen rich punctae can been seen pushing into the endothelial cell followed by a dissolution of TKS5-zsGreen and continued cell migration

**Movie 2: Invadopodia retraction *in vivo.***

**(A and B)** Representative movies of MDA-MB-231 control cell, green, forming and retracting invadopodia protrusion through the endothelium (red). Nuclei were labeled with Hoechst in **(B)**. **Movie 3: MDA-MB-231 cell protrusion and retraction velocity measurements in the chick CAM** Representative movie of the quantification of protrusion and retraction velocity in MDA-MB-231 control (**A**) and PAK1 shRNA (**B**), green, in the vasculature of the CAM (red).

**Movie 4: Detection of metastatic colonies in the CAM.** 5 days post-i.v injection of MDA-MB-231 cells the chick CAM was i.v. injected with Rhodamie-lectin and Hoechst. Metastatic colonies (green) were imaged using an upright Nikon confocal microscope form the top of the vasculature (red) down through the CAM.

**Movie 5: Generation of a chemotactic gradient in the chick CAM.** 100ng/ml FITC-EGF in Matrigel was added to the top of the CAM. 3hrs post-EGF/Matrigel addition rhodamine-lectin and Hoechst were injected to label endothelial cells (red) and nuclei (blue). Confocal microscopy imaging was performed every 3hrs to observe the distribution of FITC-EGF overtime. Movies of stacks showing the Matrigel at the top of the CAM (solid green) and diffusion through the CAM at indicated time points (**A**) 3hrs, (**B**) 6hrs, (**C**) 9hrs and (**D**) 12hrs.

**Movie 6: EGFR-GFP localization to invadopodia during the early steps of cancer cell extravasation.** MDA-MB-231 cells were transiently transfected to express human EGFR-GFP and i.v. injected into the chorioallantoic membrane of chick embryos. At various timepoints, the formation of invadopodia that breach endothelium is observed to localize EGFR-GFP.
